# Supplementary material for: Evaluation of a novel community-based COVID-19 ‘Test-to-Care’ model for low-income populations
Source: PLoS One. 2020 Oct 9;15(10):e0239400. doi: 10.1371/journal.pone.0239400 (PMC7546468; doi:10.1371/journal.pone.0239400)
Supplement: S1 Table — (DOCX) [file pone.0239400.s001.docx]

|  | Completely disagree | Disagree | Neither agree nor disagree | Agree | Completely agree |
| --- | --- | --- | --- | --- | --- |
| 1. This was an acceptable intervention for the needs of low-income Latinx individuals with COVID-19. | ➀ | ➁ | ➂ | ➃ | ➄ |
| 2. Most providers would find this intervention to be appropriate for other low-income Latinx individuals with similar needs. | ➀ | ➁ | ➂ | ➃ | ➄ |
| 3. This intervention proved effective in supporting the needs of low-income Latinx individuals with COVID-19. | ➀ | ➁ | ➂ | ➃ | ➄ |
| 4. Individuals’ needs were severe enough to warrant undertaking this intervention. | ➀ | ➁ | ➂ | ➃ | ➄ |
| 5. This intervention was a good way to address the needs of low-income Latinx individuals with COVID-19. | ➀ | ➁ | ➂ | ➃ | ➄ |
| 6. This intervention did *not* result in any unintended harms or consequences for participants. | ➀ | ➁ | ➂ | ➃ | ➄ |
| 7. I liked the procedures and approach used in this intervention. | ➀ | ➁ | ➂ | ➃ | ➄ |
| 8. I enjoyed working as a member of the Test-to-Care team. | ➀ | ➁ | ➂ | ➃ | ➄ |
| 9. This (or similar) intervention would be appropriate for a variety of low-income populations during COVID-19. | ➀ | ➁ | ➂ | ➃ | ➄ |
| 10. I would suggest the use of the Test-to-Care intervention to other providers and/or policy makers | ➀ | ➁ | ➂ | ➃ | ➄ |

S1 Table. Test-to-Care Model acceptability and appropriateness survey questions
