# Supplementary material for: Evaluation of a novel community-based COVID-19 ‘Test-to-Care’ model for low-income populations
Source: PLoS One. 2020 Oct 9;15(10):e0239400. doi: 10.1371/journal.pone.0239400 (PMC7546468; doi:10.1371/journal.pone.0239400)
Supplement: S2 Table — (DOCX) [file pone.0239400.s002.docx]

S2 Table. Test-to-Care Model feasibility survey questions

|  | Completely disagree | Disagree | Neither agree nor disagree | Agree | Completely agree |
| --- | --- | --- | --- | --- | --- |
| 1. The Test-to-Care Model seems implementable in other settings. | ➀ | ➁ | ➂ | ➃ | ➄ |
| 2. The Test-to-Care Model seems possible to carry out. | ➀ | ➁ | ➂ | ➃ | ➄ |
| 3. The Test-to-Care Model seems doable for other providers and community health workers to undertake. | ➀ | ➁ | ➂ | ➃ | ➄ |
| 4. The Test-to-Care Model seems easy to carry out. | ➀ | ➁ | ➂ | ➃ | ➄ |
| 5. The Test-to-Care Model could be integrated within the existing public health infrastructure. | ➀ | ➁ | ➂ | ➃ | ➄ |
| 5. If implemented, the Test-to-Care Model seems sustainable. | ➀ | ➁ | ➂ | ➃ | ➄ |
| 6. Providers and/or policy makers in other settings would likely be interested/excited about implementing a similar model to address the needs of low-income individuals with COVID-19. | ➀ | ➁ | ➂ | ➃ | ➄ |
| 7. The Test-to-Care Model could be adapted to different settings to better serve the needs of local communities. | ➀ | ➁ | ➂ | ➃ | ➄ |
