# Supplementary material for: Evaluation of a novel community-based COVID-19 ‘Test-to-Care’ model for low-income populations
Source: PLoS One. 2020 Oct 9;15(10):e0239400. doi: 10.1371/journal.pone.0239400 (PMC7546468; doi:10.1371/journal.pone.0239400)
Supplement: S3 Table — Providers and community health workers completed a brief web-based survey of 17, five-point Likert scale questions, where 1 = Strongly disagree, 2 = Disagree, 3 = Neither Agree nor Disagree, 4 = Agree, and 5 = Strongly Disagree. Therefore, the higher the mean value, the more acceptable/feasible the Test-to-Care Model was felt to be. All mean values were out a possible score of 5. (DOCX) [file pone.0239400.s003.docx]

S3 Table. The acceptability and feasibility of the Test-to-Care according to providers (n=5) and community health workers (n=2) (n=7 total). Providers and community health workers completed a brief web-based survey of 17, five-point Likert scale questions, where 1=Strongly disagree, 2=Disagree, 3=Neither Agree nor Disagree, 4=Agree, and 5=Strongly Disagree. Therefore, the higher the mean value, the more acceptable/feasible the Test-to-Care Model was felt to be. All mean values were out a possible score of 5.

|  | **Mean (SD)** | **Proportion who agree or strongly agree** |
| --- | --- | --- |
| **Acceptability and appropriateness*** |  |  |
| 1. This was an acceptable intervention for the needs of low-income Latinx individuals with COVID-19. | 4.0 (0.6) | 86 |
| 2. Most providers would find this intervention to be appropriate for other low-income Latinx individuals with similar needs. | 4.3 (0.5) | 100 |
| 3. This intervention proved effective in supporting the needs of low-income Latinx individuals with COVID-19. | 3.7 (1.0) | 71 |
| 4. Individuals’ needs were severe enough to warrant undertaking this intervention. | 4.6 (0.5) | 100 |
| 5. This intervention was a good way to address the needs of low-income Latinx individuals with COVID-19. | 4.0 (0.8) | 71 |
| 6. This intervention did *not* result in any unintended harms or consequences for participants. | 4.1 (1.0) | 57 |
| 7. I liked the procedures and approach used in this intervention. | 4.0 (0.6) | 86 |
| 8. I enjoyed working as a member of the Test-to-Care team. | 4.4 (0.8) | 86 |
| 9. This (or similar) intervention would be appropriate for a variety of low-income populations during COVID-19. | 4.4 (0.8) | 100 |
| 10. I would suggest the use of the Test-to-Care intervention to other providers and/or policy makers | 4.1 (0.7) | 86 |
| **Feasibility*** |  |  |
| 1. The Test-to-Care Model seems implementable in other settings. | 4.4 (0.5) | 100 |
| 2. The Test-to-Care Model seems possible to carry out. | 4.7 (0.5) | 100 |
| 3. The Test-to-Care Model seems doable for other providers and community health workers to undertake. | 4.7 (0.5) | 100 |
| 4. The Test-to-Care Model seems easy to carry out. | 3.4 (0.8) | 29 |
| 5. The Test-to-Care Model could be integrated within the existing public health infrastructure. | 3.9 (0.9) | 86 |
| 6. If implemented, the Test-to-Care Model seems sustainable. | 4.3 (0.5) | 100 |
| 7. Providers and/or policy makers in other settings would likely be interested/excited about implementing a similar model to address the needs of low-income individuals with COVID-19. | 4.7 (0.5) | 100 |

*The overall mean composite acceptability (4.4 vs. 4.1) and feasibility score (4.5 and 4.2) did not appear to substantially differ between CHWs and providers, respectively.
