# Supplementary material for: Evaluation of a novel community-based COVID-19 ‘Test-to-Care’ model for low-income populations
Source: PLoS One. 2020 Oct 9;15(10):e0239400. doi: 10.1371/journal.pone.0239400 (PMC7546468; doi:10.1371/journal.pone.0239400)
Supplement: S1 Appendix — (DOCX) [file pone.0239400.s004.docx]

**linkage to care**

- The initial disclosure call is used to assess and classify the overall degree of support a positive patient will receive from the T2C clinic-based team. Patients are classified into three group as follows:
  - LOW: Asymptomatic patients who are overall healthy (no underlying comorbid conditions) and are connected to primary care. These patients are able to isolate at home, have access to food and supplies, and express no overt needs.
  - MEDIUM: Patients who are asymptomatic but need some support or have underlying health conditions. This group also contains patients who have mild symptoms but are otherwise healthy.
  - HIGH: These patients need a high level of support. They are either symptomatic and/or have comorbid conditions and/or are at risk for severe symptoms and/or are not connected to care and/or have difficulty isolating in home setting and/or have barriers to food access.
- This classification will be used to determine the frequency of follow-up wellness phone calls by T2C RNs:
  - Low: every 4-7 days
  - Medium: every 3-4 days
  - High: every 1-2 days

**Note – this classification does not impact the frequency of phone calls and follow-up by T2C community health workers.*

- T2C Provider Lead assigns follow up assignments daily to T2C RNs and T2C Social Workers. T2C RNs conduct symptom assessment and telephone triage and T2C Social Workers provide linkage to care or address food insecurity.
- T2C Providers follow positive patients according to the frequency indicated until 14 days from the date of positive test result, and document ongoing follow up on Form A.
- For patients who develop new or worsening symptoms: T2C will refer patients to T2C RN for triage and patients will be given a specific COVID-19 clinic phone number to utilize during T2C follow up period.

**Form A: SCRIPT FOR DISCLOURE AND PATIENT CASE REPORT FORM**

Hi, I am calling for (patient name) [*Verify you are speaking to the correct person.*]

My name is (your name) and I am calling from ______. Can I verify your date of birth to make sure I’m talking with the right person? [*Verify DOB*]. I’m calling about the COVID-19 testing you recently had done. [*In a calm voice, inform the patient of the following*:] The result of your COVID-19 test was positive. [*To clarify, say:*] This means that you were recently infected with COVID-19. [*Pause, then ask*] How are you feeling about this? [*Respond as appropriate*]. I have to ask you several questions, and I will provide you with some information around ways to keep yourself and others safe. Do you have any questions for me before we start? [*Respond as appropriate, then proceed with questions below*].

**Symptoms and Medical History**:

Have you had any new or resolved symptoms of COVID-19? (See Form D for symptom list)

| NO | YES | Symptoms: |
| --- | --- | --- |
|  |  | Duration: |
|  |  | Past/resolved symptoms: |

Do you have any underlying medical conditions? (See Form D for symptom list)

| NO | YES | Chronic medical conditions: |
| --- | --- | --- |

**Housing:** Do you live in an apartment or a house? Are you unhoused? Are you able to isolate away from others in your current living situation? (If no, See form B; I & Q Housing)

| Unhoused | Housed, able to isolate. | Housed, Unable to isolate: |
| --- | --- | --- |
|  |  | Type of home: |
|  |  | Number of people in household/names/age: |
|  |  | Shared space details (private bath?): |

**Employment**: Are you currently working outside of your home? If so, we advise you to stop working around others outside the home now. I have a few questions about your work (see Isolation information below for duration):

| No | Yes | Needs work *excuse* letter | Needs *return* to work letter |
| --- | --- | --- | --- |
|  | Details/occupation: | Date range: | RTW date: |
|  |  | Date sent to patient: | Date sent to patient: |

**Resources for isolation**: Is someone able to drop off or help you get food, masks, and other resources you may need to isolate at home for 10-14 days? If not, notify the patient that a T2C Social Worker will be reaching out to them by phone within 24 hours to talk about support and resources for people experiencing hardships due to COVID-19 (211 for food, rent resources, unemployment resources).

| Specific social work needs/follow up items: |  |
| --- | --- |

**General Isolation information**: (as of 5/14/2020 – this information should be updated per most current CDC guidelines) Advise patient, if COVID-19 positive test and asymptomatic, isolate for 10 days from day of positive test. If COVID-19 positive and symptomatic, isolate while symptomatic and 72 hours after symptoms resolve. If isolating at home with others, move to single room if possible. Use a mask as all times around others, clean bathroom after use, frequent hand washing. We will continue to call you for follow up for the next 14 days.

**Follow up classification: circle one**

| High  (daily calls x14 days) | Medium  (calls every 3-4 days x14 days) | Low  (calls every 4-7 days x14 days) |
| --- | --- | --- |

**Follow up Notes:**

|  |
| --- |

**FORM B: SCRIPT FOR ISOLATION INSTRUCTIONS**

The initial disclosure call is used to assess and classify the **FORM B: SCRIPT FOR ISOLATION INSTRUCTIONS**

"Because your COVID-19 test was positive, you will need to isolate yourself from other members of your household and the general public for the next 10 days. You should wear a face covering at home when you are around other people. If you are not able to wear a face covering, then people who live with you should wear a face covering when they are in the same room with you **(if children live in home, see special considerations below)**. You are not allowed to go out for anything but essential medical visits. If possible, you should get someone else to do your shopping for you and tell your friend, neighbor, family member, or delivery service leave your food, medicine, or essential items on the doorstep. You may have many questions about this, and I am happy to answer as many as I can now.

**Special considerations for masks and children: Use masks and face covers with caution with children. Infants and children under 2 should not wear face coverings. Those between the ages of 2 and 8 should use them but under adult supervision to ensure that the child can breathe safely and avoid choking or suffocation. Children with breathing problems should not wear a face covering.**

**FORM C: ISOLATION and QUARANTINE HOTEL PLACEMENT REFERRAL**

When the T2C provider assesses that the patient is not able to isolate from family/others in household during the disclosure call, the questions below must be asked in preparation for the I&Q housing referral.

If the patient is unable to isolate at home and open to placement in a free isolation hotel provided by the City, please inform the patient that:

- They will be staying in a hotel room with a TV and private bathroom for up to 7-14 days.
- They will be expected to stay inside the room the entire time.
- Meals will be delivered 3 times a day but they cannot order room service, as this hotel is staffed by nurses, not hotel staff.
- Nurses are available 24/7 to assist with any medical needs or questions.

Ask the patient:

1. Do you have any ADA needs? For example, do you use a wheelchair?
2. Will you have to bring a family member with you if you move to isolation in a hotel room? If so, obtain family member’s name, DOB, MRN (if applicable), and contact phone number.
3. Do you have a pet that you will bring with you to the isolation hotel?
4. Do you have a bicycle that you will bring with you to the isolation hotel?
5. Do you have any dietary needs?
6. Do you have a case manager? If so, obtain name of case manager and contact phone number.
7. Do you take methadone and/or suboxone?
8. Do you take any medication for mental health?

.

**FORM d: SCREENING FOR COVID-19 SYMPTOMS**

**RISK FACTORS – INITIAL CALL**

| **CHRONIC CONDITIONS**   - Generally Healthy - Diabetes I - Diabetes II - Cardiovascular condition(s) - Pulmonary condition(s) - Chronic kidney disease - Cancer (in chemo/radiation) - Cancer (remission) - Autoimmune disease - Other condition:   **IMMUNOSUPPRESSIVE THERAPY**   - Yes - No - No information | **SMOKING**   - Yes, tobacco - Yes, marijuana - Yes, vaping - No - Prior smoker - No information   **PREGNANCY?**   - Yes - No - No information   **RISK STRATIFICATION**   - High risk (>50 with risk factors or >70 with or without risk factors) - Medium risk (>50 without risk factors or <50 with risk factors) - Low risk (<50 without risk factors) |
| --- | --- |

**SYMPTOM SCREEN, CLINICAL CLASSIFICATION, RECOMMENDATIONS – INITIAL AND FOLLOW UP CALLS**

| **SYMPTOM SCREEN**   - Tested for COVID-19 Previously - New fever (>37.8°C/100.0°F) - Shortness of breath at rest - Shortness of breath on exertion - Sharp pain in chest when breathing - Dry cough - Cough with phlegm - Sore throat - Drenching night sweats - Shaking chills - Body aches - Unexplained fatigue - Unexplained headache - Diarrhea - Loss of smell or taste - Sinus congestion - Sneezing or runny nose different from underlying allergies - Feeling better than during last call - Feeling about the same as during last call - Feeling worse than during last call | **CLINICAL CLASSIFICATION**   - LOW (mild symptoms and not worsening) - MEDIUM (Not markedly better than previous call) - HIGH (Potentially worsening) - RECOMMEND ASSESSMENT (markedly worsening symptoms; fever for 5+ days; shortness of breath) |
| --- | --- |
| **RECOMMENDATIONS**   - Fluid intake - Nutritional intake - Breathing exercises - Light exercise at home if able - Symptom management (pain, fever, nausea) - Motivation and encouragement - Referral to information sources - Referral to T2C social work |  |
